# Supplementary material for: Pancreatic cancer-associated fibroblasts modulate macrophage differentiation via sialic acid-Siglec interactions
Source: Commun Biol. 2024 Apr 9;7:430. doi: 10.1038/s42003-024-06087-8 (PMC11003967; doi:10.1038/s42003-024-06087-8)
Supplement: Supplementary file 2 — Supplementary information [file 42003_2024_6087_MOESM2_ESM.pdf]

Supplementary Information

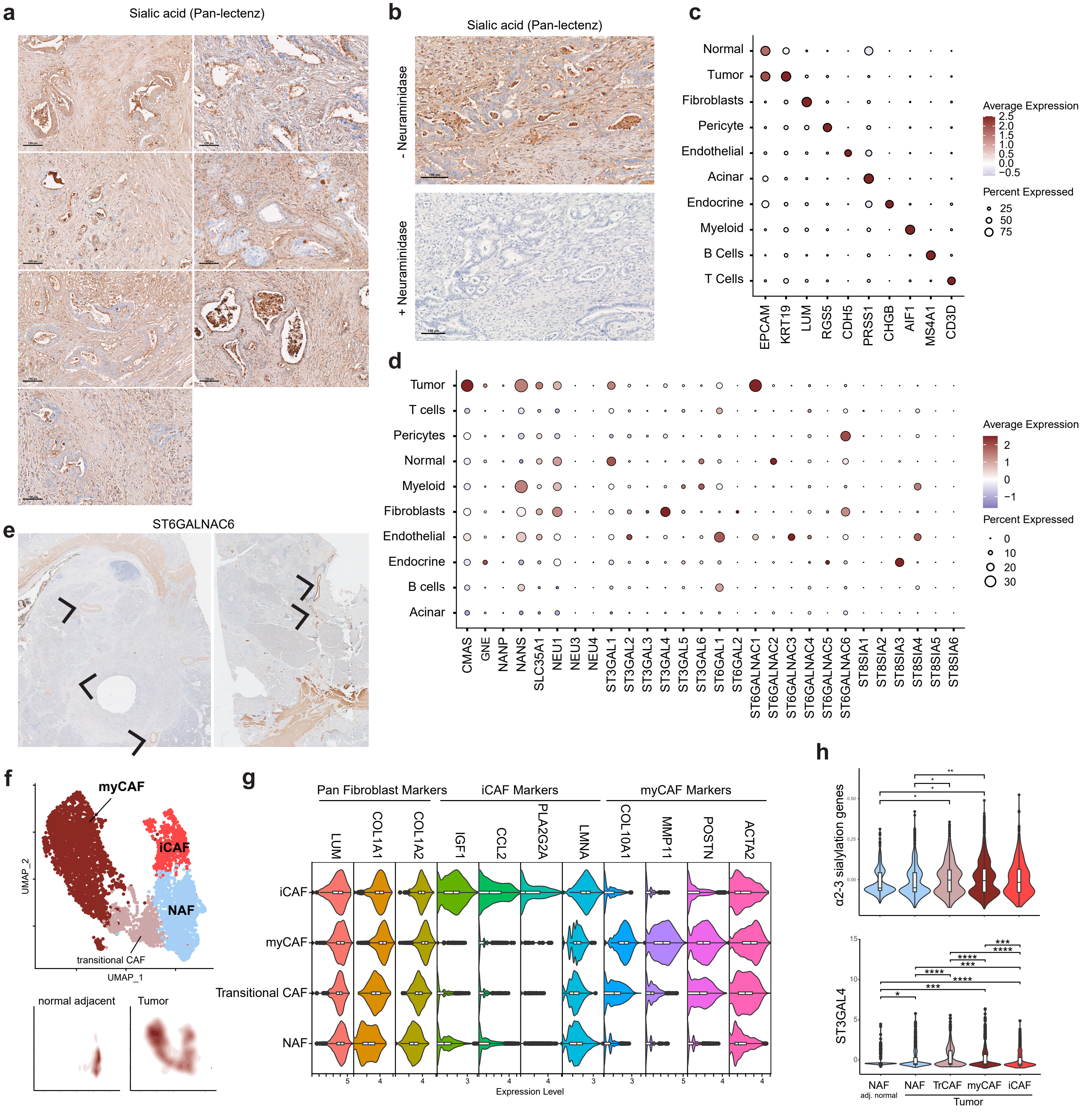

Supplementary Figure 1, associated with Figure 1. a) Immunohistochemical staining of sialic acid expression using pan-Lectenz in PDAC patient biopsies, with each image representing an individual patient. Scale bar equals 100 µm. b) Immunohistochemical staining for sialic acid expression using pan-Lectenz after treatment of the tissue with neuraminidase. Scale bar equals 100 µm. c) Bubble plot showing cell type–specific markers across all clusters in PDAC scRNA-seq dataset from Peng et al. [1]. d) Bubble plot showing sialylation related gene expression across all clusters in PDAC scRNA-seq dataset from Peng et al. [1]. e) Immunohistochemical staining of the sialyltransferase ST6GALNAC6 in PDAC biopsies (n = 3). Black arrows indicate ST6GALNAC6 expression surrounding vessel or in duct-like structures. f) UMAP illustrating the integration and re-clustering of fibroblasts from scRNA-seq dataset from Peng et al. [1]. Upper UMAP illustrates clustering that revealed 4 fibroblast subsets: normal-associated fibroblasts (NAF), inflammatory CAFs (iCAF), transitional CAFs, and myofibroblastic CAFs (myCAF). Bottom plots show the density of cells derived from normal or tumor samples across the UMAP. g) Expression of CAF-related markers per CAF subsets from (f). h) Gene score for α2-3 sialylation, and ST3GAL4 expression in CAF subsets from (f). Statistical analysis of all pairwise comparisons was done using the Wilcoxon test, with Holm's correction for multiple comparison test. Only significative results are shown. Data presented as boxplot indicate the median, 25th and 75th percentiles (hinges) and whiskers represent 1.5 times the interquantile range.

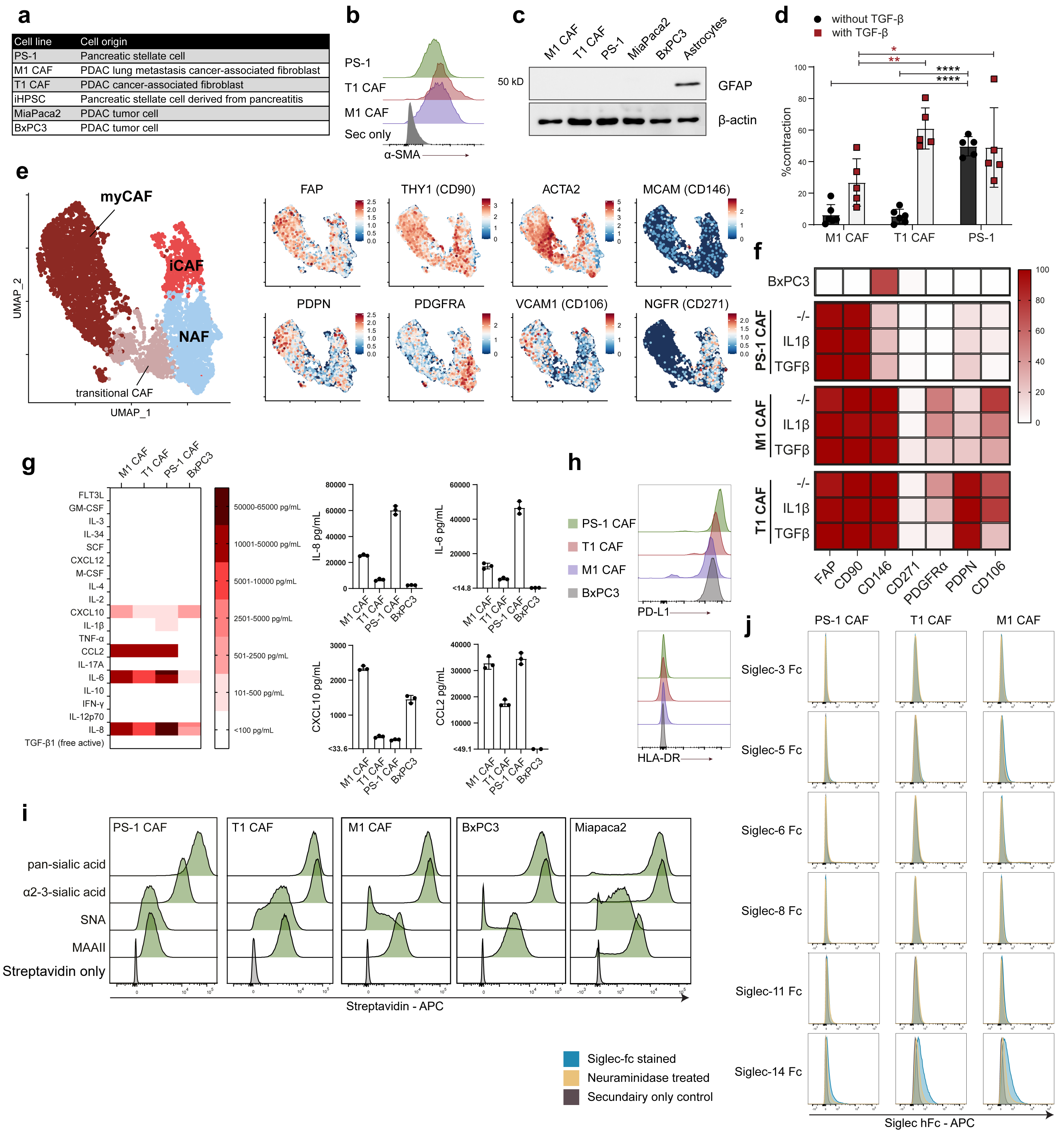

Supplementary Figure 2: associated with Figure 2. a) Table of the cell lines used in this paper. b) Expression of α-SMA in fibroblast cell lines assessed by flow cytometry, compared to the negative control stained with secondary antibody only. c) Westernblot for GFAP protein in the fibroblast cell lines, tumor cell lines as negative control and astrocytes as positive control. d) Collagen gell contraction assay showing percentage contraction of fibroblast cell lines under steady culture state, or treated with TGF-β as positive control. Statistical analysis using two-way ANOVA and Tukey's multiple comparisons test, comparing cell lines within each group. e) UMAP of scRNA-seq dataset of Peng et al.[1] showing expression of several genes across fibroblast cell clusters. f) Expression of fibroblast markers on the CAF cell lines in steady state or after stimulation with IL-1β or TGF-β. BxPC3 tumor cells were included as negative control. g) Heatmap of cytokines and chemokines secreted by the tumor cell line BxPC3 and CAF cell lines. Specific levels of the most highly secreted cytokines and chemokines are shown per cell line. h) Histograms of PD-L1 and HLA-DR expression in BxPC3 and CAF cell lines evaluated by flow cytometry. i) Histograms belonging to Figure 2B-C, illustrating sialic acid expression on the different cell lines. j) Flow cytometric analysis of Siglec ligand expression in CAF cell lines treated with/without neuraminidase.

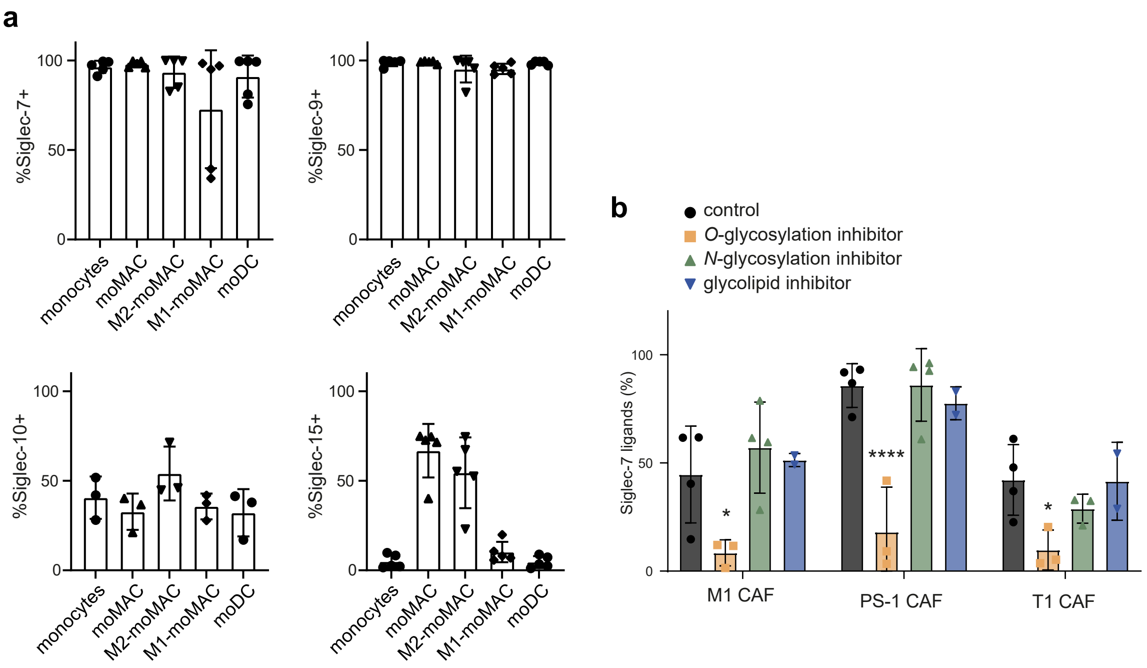

Supplementary Figure 3. a) Siglec receptor expression on in vitro differentiated moMACs and moDCs, analyzed by flow cytometry. b) Percentage Siglec-7 ligand positive cells after treatment of CAF cell lines with several glycosylation inhibitors, evaluated by flow cytometry. Statistical analysis using two-way ANOVA and dunnett's multiple comparison test, comparing individual glycan inhibitor conditions to control within each cell line.

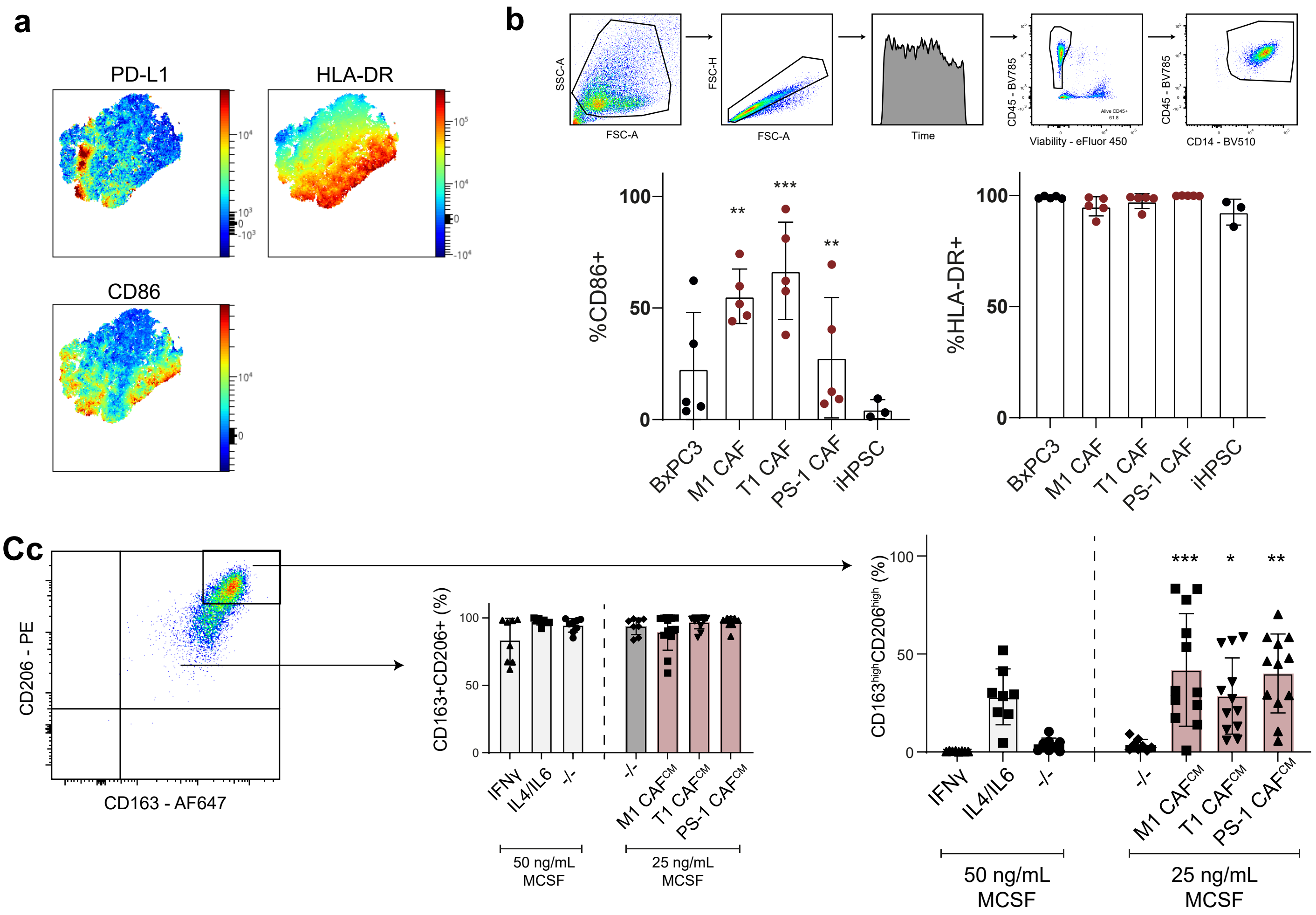

Supplementary Figure 4: associated with Figure 4. a) Expression of PD-L1, HLA-DR, CD86 across the tSNE belonging to Figure 4b. b) Percentage of CD86 and HLA-DR positive cells after co-culture with BxPC3 and CAF cell lines, gated from CD45+CD14+ cells. c) Differentiation of monocytes in the presence of CAF-conditioned media (CM) evaluated at day 4. Statistical analysis with one-way ANOVA and dunnett's multiple comparison test comparing conditioned media vs 25 ng/mL MCSF only (-/-).

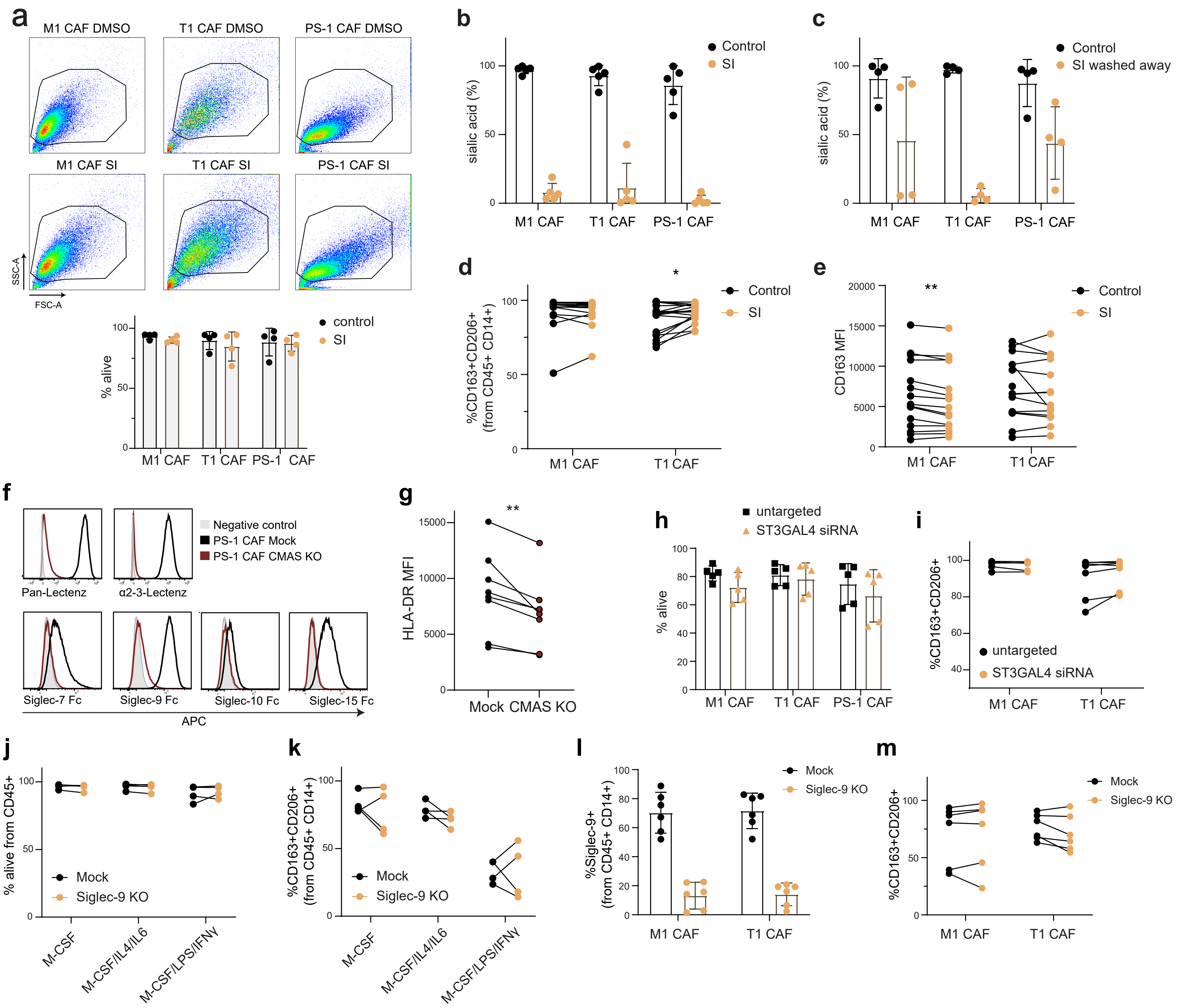

Supplementary Figure 5: associated with Figure 5. a) Forward and side scatter of CAF cell lines treated with sialylinhibitor or DMSO, and cell viability of CAF cell lines after 4 day treatment with sialylinhibitor (SI) or DMSO. Evaluated by flow cytometry. b) Percentage of sialic acid positive CAFs using Siglec-9-Fc after treatment with 200 μM SI for 4 days. c) Percentage of sialic acid positive CAFs using Siglec-9-Fc on day 3 after removing the SI from the culture. d) Percentage of CD163+CD206+ macrophages after co-culture with T1 and M1 CAFs, treated with SI or DMSO control prior to co-culture. e) CD163 expression on CD14+ cells after co-culture with the M1 CAF or T1 CAF cell lines treated with SI or DMSO control prior to co-culture. f) Crispr-CAS9 KO of the CMAS gene in PS-1 CAF results in a depletion of sialylated glycans and Siglec ligands on the PS-1 cell line, as evaluated by flow cytometry staining using pan-Lectenz, α2-3-Lectenz, or Siglec-7, -9, -10 and -15-Fcs. Black histogram represents PS-1 CAF Mock transfected, red line is PS-1 CAF CMAS KO, gray histogram represents the negative secondary only control. g) Expression of HLA-DR on CD45+CD14+ cells after co-culture with PS-1 CAF mock transfected or PS-1 CAF CMAS KO. h) Cell viability of the CAF cell lines after knockdown of ST3GAL4 using siRNA. i) Percentage of CD163+CD206+ macrophages after co-culture with M1 CAFs or T1 CAFs, knocked-down for ST3GAL4 prior to co-culture. j) Cell viability of macrophages after in vitro differentiation of Siglec-9 KO or mock transfected monocytes. k) Percentage CD163+CD206+ macrophages after in vitro differentiation of Siglec-9 KO or mock transfected monocytes. l) Percentage of Siglec-9+ cells within CD14+ monocytes on day 4 of co-culture with CAF cell lines after transfection of monocytes with Siglec-9 KO plasmid or Mock plasmid. m) Differentiation of Mock and Siglec-9 KO monocytes after co-culture with M1 CAFs or T1 CAFs. %CD163+CD206+ cells were gated within CD14+ cells for Mock transfected monocytes and gated on the CD14+ Siglec-9- population for the Siglec-9 KO transfected monocytes.

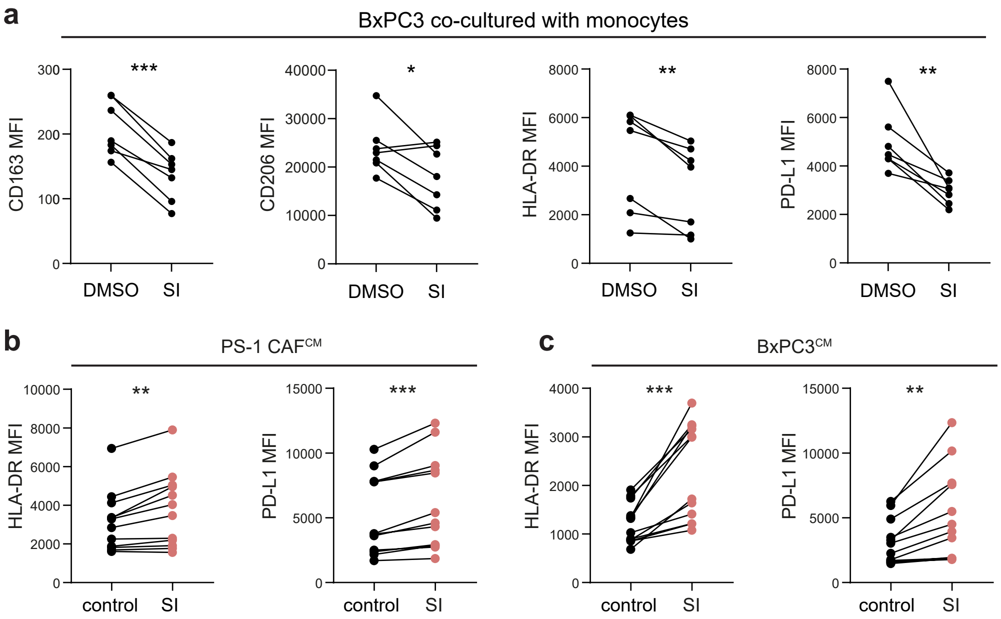

Supplementary Figure 6: associated with Figure 6. a) Expression of CD163, CD206, HLA-DR and PD-L1 on CD14+ macrophages after co-culture with BxPC3, treated with SI or DMSO control prior to co-culture. b) Expression of HLA-DR and PD-L1 on monocytes differentiated in the presence of conditioned media (CM), derived from PS-1 CAF treated with SI or DMSO control. c) Expression of HLA-DR and PD-L1 on monocytes differentiated in the presence of conditioned media (CM), derived from BxPC3 treated with SI or DMSO control.

Supplementary Table 1: Stage of lesions for samples included in tissue stainings

pTN stage
pT2N2
pT2N0
pT2N0
pT3N2
pT2N2
pT3N1
pT1cN1
pT2N2
pT2N1
pT2N0

Supplementary Table 2: List of antibodies used in this study

Antibody	dilution	source	Cat number
Macrophage differentiation/polarisation panel - 15 color Aurora			
LIFE/DEAD BLUE	1/2000	Thermofisher	L23105
anti-CD163 bv421	1/200	Biolegend	333612
anti-CD14 AF700	1/25	Biolegend	301842
anti-CD86 bv650	1/800	Biolegend	305428
anti-CD45 BUV805	1/50	BD biosciences	612892
anti-DC-SIGN (AZN-D1) AF488	1/800	in house	
anti-CD206 PE-cy5	1/800	Biolegend	321106
anti-PD-L1 PE-cy7	1/800	Biolegend	329718
anti-CD1a APC-fire750	1/800	Biolegend	300142
anti-HLA-DR BUV563	1/800	BD biosciences	748340
anti-Siglec-7 Percp-vio700	1/50	Miltenyi Biotec	130-100-979
anti-siglec9 AF594	1/100	R&D systems	FAB1139T
anti-Siglec-15	1/1000	Novus	NBP2-41162
anti-rabbit IgG AF647	1/800	Invitrogen	A-31573
anti-Siglec-10 BV786	1/50	BD biosciences	747709
Macrophage differentiation/polarisation panel - 7 color Fortessa			
viability dye eFluor450	1/1000	ebiosciences	65-0863-14
anti-CD14 bv510	1/25	Biolegend	301842
anti-CD86 bv650	1/50	Biolegend	305428
anti-CD45 bv786	1/100	Biolegend	304048
anti-CD206 PE	1/50	Biolegend	321106
anti-PD-L1 PE-cy7	1/200	Biolegend	329718
anti-CD163 AF647	1/200	Biolegend	333620
anti-HLA-DR AF700	1/75	BD biosciences	560743
Other antibodies or reagents for flow cytometry			
human Fc block	1/500	BD biosciences	564220
True-stain monocyte blocker	1/100	Biolegend	426103
Brilliant stain buffer	1/2	BD biosciences	566349
Streptavadin APC	1/400	BD biosciences	554067
anti- α-SMA unlabeled	1/100	DAKO	M085129-2
Fibroblast markers			
anti-CD90 bv785	1/200	Biolegend	328142
anti-FAP AF488	1/25	R&D Systems	FAB3715G
anti-PD-L1 PE	1/25	Biolegend	329706
anti-HLA-DR PE-Fire810	1/200	Biolegend, custom conjugated	clone L243
anti-PDPN AF647	1/50	Biolegend	337008
anti-CD34 PE-cy7	1/100	Biolegend	343515
anti-CD146 BV711	1/100	Biolegend	361031
anti-CD271 PerCP-cy5.5	1/100	Biolegend	345111
anti-PDGFRa APC	1/50	Biolegend	323511
anti-CD106 BV421	1/200	Biolegend	305815
anti- αSMA	1/100	DAKO	M085129-2
Antibodies for ELISA			
Capture antibody - IL-10	1/1000	eBioscience	14-7108-85
Detection antibody - IL-10	1/1000	eBioscience	13-7109-85
Antibodies for Multiplex Microscopy			
ST3GAL4	1/50	Sigma Aldrich	HPA049827
ST6GALNAC6	1/10	Sigma Aldrich	HPA018890
a-SMA	1/1000	DAKO	M085129-2
panCK	1/1000	Abcam	ab86734
CD45	1/500	DAKO	M070101-2
Siglec-7	1/400	Invitrogen	PA5-115899
Siglec-9	1/50	Atlas Antibodies	HPA010682
Siglec-10	1/50	Thermofisher	PA5-55501
Siglec-15	1/8000	Thermofisher	PA5-116878
CD14	1/200	Abcam	ab13335
CD163	1/300	Invitrogen	MA5-11458

Supplementary Table 3: List of gene sets.

Fibroblasts	Tumor	Donor Synthesis	2.3 Sialylation	2.8 Sialylation	2.6 - Gal Sialylation	2.6 - GalNAc Sialylation	Sialylation All
LUM	TFF1	GNE	ST3GAL1	ST8SIA1	ST6GAL1	ST6GALNAC1	ST6GALNAC1
SFRP2	FXYD3	NANS	ST3GAL2	ST8SIA2	ST6GAL2	ST6GALNAC2	ST6GALNAC2
COL1A1	TFF3	NANP	ST3GAL3	ST8SIA3		ST6GALNAC3	ST6GALNAC3
APOD	TFF2	CMAS	ST3GAL4	ST8SIA4		ST6GALNAC4	ST6GALNAC4
DCN	KRT19	SLC35A1	ST3GAL5	ST8SIA5		ST6GALNAC5	ST6GALNAC5
COL1A2	LCN2		ST3GAL6			ST6GALNAC6	ST6GALNAC6
COL3A1	C19orf33						ST3GAL1
CTHRC1	S100A6						ST3GAL2
FN1	REG4						ST3GAL3
PTGDS	AGR2						ST3GAL4
FBLN1	CEACAM6						ST3GAL5
SFRP4	LGALS4						ST3GAL6
CXCL14	S100P						ST6GAL1
CCDC80	SLPI						ST6GAL2
MGP	PHGR1						GNE
COL6A3	MUC1						NANS
MMP11	C15orf48						NANP
SERPINF1	WFDC2						CMAS
C7	CTSE						SLC35A1
CYR61	TSPAN8						ST8SIA1
C1S	PDZK1IP1						ST8SIA2
CFD	CLDN18						ST8SIA3
IGFBP5	GPX2						ST8SIA4
AEBP1	SPINK1						ST8SIA5
CTGF	MMP7						
RARRES2	CLDN4						
C1R	SMIM22						
SPARC	SERPINA1						
BGN	OLFM4						
MMP2	SPINT2						
POSTN	KRT7						
COL6A2	LGALS3						
COL10A1	TSPAN1						
TIMP1	LSR						
VCAN	ELF3						
IGFBP3	S100A14						
LGALS1	LYZ						
CTSK	PGC						
COL5A2	PIGR						
ASPN	KRT8						
THBS2	CYSTM1						
MFAP4	PI3						
PCOLCE	TMC5						
COL6A1	GPRC5A						
C3	OCIAD2						
NBL1	S100A11						
INHBA	CEACAM5						
CALD1	TACSTD2						
THY1	MAL2						
COL11A1	KRT18						

References

1. Peng, J., et al., *Single-cell RNA-seq highlights intra-tumoral heterogeneity and malignant progression in pancreatic ductal adenocarcinoma.* Cell Res, 2019. **29**(9): p. 725-738.
