# Supplementary material for: Pancreatic cancer-associated fibroblasts modulate macrophage differentiation via sialic acid-Siglec interactions
Source: Commun Biol. 2024 Apr 9;7:430. doi: 10.1038/s42003-024-06087-8 (PMC11003967; doi:10.1038/s42003-024-06087-8)
Supplement: Supplementary file 3 — Description of Additional Supplementary Files [file 42003_2024_6087_MOESM3_ESM.pdf]

Description of Additional Supplementary Files

File name: Supplementary data 1

Description: This table includes the source data belonging to figure 3a, where the measurements for each specific glycan can be found per cell line.
